# Oligo-Fucoidan Improves Diabetes-Induced Renal Fibrosis via Activation of Sirt-1, GLP-1R, and Nrf2/HO-1: An In Vitro and In Vivo Study

**DOI:** 10.3390/nu12103068

**Published:** 2020-10-08

**Authors:** Wen-Chun Yu, Ren-Yeong Huang, Tz-Chong Chou

**Affiliations:** 1 Graduate Institute of Life Sciences, National Defense Medical Center, Taipei 11490, Taiwan; jushisa1114@gmail.com; 2 Department of Periodontology, School of Dentistry, Tri-Service General Hospital, National Defense Medical Center, Taipei 11490, Taiwan; ndmcandy@ndmctsgh.edu.tw; 3 Graduate Institute of Medical Sciences, National Defense Medical Center, Taipei 11490, Taiwan; 4 Department of Pharmacology, National Defense Medical Center, Taipei 11490, Taiwan; 5 China Medical University Hospital, China Medical University, Taichung 404332, Taiwan; 6 Department of Biotechnology, Asia University, Taichung 41354, Taiwan; 7 Cathay Medical Research Institute, Cathay General Hospital, New Taipei City 22174, Taiwan

**Keywords:** fucoidan, renal fibrosis, diabetes, transforming growth factor-β, Sirt-1, glucagon-like peptide-1 receptor

## Abstract

Fucoidan extracted from brown algae has multiple beneficial functions. In this study, we investigated the effects of low-molecular-weight fucoidan (oligo-FO) on renal fibrosis under in vitro and in vivo diabetic conditions, and its molecular mechanisms. Advanced glycation product (AGE)-stimulated rat renal proximal tubular epithelial cells (NRK-52E) and diabetic mice induced by high-fat diet and intraperitoneal injection of streptozotocin and nicotinamide were used. Oligo-FO treatment significantly inhibited anti-high mobility group box 1 (HMGB1)/RAGE/ anti-nuclear factor-kappa B (NF-κB)/transforming growth factor-β1 (TGF-β1)/TGF-β1R/Smad 2/3/fibronectin signaling pathway and HIF-1α activation in AGE-stimulated NRK-52E cells. Conversely, the expression and activity of Sirt-1; the levels of ubiquitin-specific peptidase 22 (USP22), *p*-AMPK, glucagon-like peptide-1 receptor (GLP-1R), and heme oxygenase-1 (HO-1); and Nrf2 activation were remarkably increased by oligo-FO in AGE-stimulated cells. However, the above effects of oligo-FO were greatly diminished by inhibiting Sirt-1, HO-1, or GLP-1R activity. Similar changes of these pro-fibrotic genes in the kidney and a marked attenuation of renal injury and dysfunction were observed in oligo-FO-treated diabetic mice. These findings indicated that the inhibitory effects of the oligo-FO on diabetes-evoked renal fibrosis are mediated by suppressing TGF-β1-activated pro-fibrogenic processes via Sirt-1, HO-1, and GLP-1R dependence. Collectively, fucoidan-containing foods or supplements may be potential agents for ameliorating renal diseases due to excessive fibrosis.

## 1. Introduction

The diabetic nephropathy (DN), a severe vascular complication in diabetic patients, is considered to be a leading factor causing end-stage renal disease [1]. The major pathological features of DN include glomerular mesangial cell (GMC) proliferation and hypertrophy, extracellular matrix (ECM) accumulation, glomerulosclerosis, and tubulointerstitial fibrosis [2]. To date, the true mechanisms causing the characterized symptoms of DN, especially renal fibrosis, remain unclear. Growing evidence has suggested that transforming growth factor-β (TGF-β)/Smad-induced generation of ECM components such as fibronectin (FN) and collagens plays a critical role in triggering renal fibrosis associated with DN [3]. Although current drugs and advanced therapies have been used to treat DN, the outcomes are still poor [4]. Thus, development of new agents and functional foods that have an ability to inhibit TGF-β-mediated processes may be an effective strategy to alleviate DN.

Sirt-1, a nicotinamide adenosine dinucleotide (NAD)-dependent deacetylase, has several cellular and physiological functions, including regulation of glucose and lipid metabolism, inflammatory responses, and insulin secretion [5]. Treatment with Sirt-1 agonists or activation of Sirt-1 has been confirmed to reduce renal cell apoptosis and fibrosis by inhibiting TGF-β/Smad cascade and ERK1/2-regulated processes [6]. Conversely, severe albuminuria and mitochondrial dysfunction were observed in Sirt-1 knockdown diabetic mine [7]. Accordingly, enhancing Sirt-1 expression/activity has become an attractive target to ameliorate renal damage and fibrosis during DN.

The nuclear factor erythroid-2-related factor 2 (Nrf2) is a transcriptional factor, and exhibits a protective effect against oxidative stress-associated diseases [8]. Under oxidative insult, Nrf2 dissociates from the repressor protein Kelch-like ECH-associated protein 1 (Keap1), and eventually leads to Nrf2 nuclear translocation and activation, thereby activating downstream phase II antioxidant gene transcription, including superoxide dismutase and heme oxygenase-1 (HO-1) [9]. In addition, Nrf2 is capable of improving diabetic renal fibrosis through downregulation of FN and intercellular adhesion molecule-1 [10]. It has been reported that the inhibitory effects of Sirt-1 on reactive oxygen species (ROS) production and the expression of FN and TGF-β in advanced glycation products (AGEs)-stimulated GMCs were also mediated by activation of Nrf2 [11].

Glucagon-like peptide-1 (GLP-1), an incretin, has multiple protective effects. A previous study has indicated that the anti-hyperglycaemic activity of GLP-1 and GLP-1 receptor (GLP-1R) cascade is associated with enhancing insulin secretion and attenuating pancreatic β-cell apoptosis [12]. Based on these findings (that administration of GLP-1R agonists remarkably attenuated DN progression both in diabetic animal and patients [13,14]), activation of GLP-1R-dependent responses is a promising target for DN therapy. Our and other studies revealed that a marked decrease in Sirt-1, GLP-1R, and Nrf2/HO-1 levels was observed in diabetic animal [15,16]. Therefore, elevation of these protective gene expressions and activation of their regulated responses are critical for attenuating DN progression. 

Fucoidan mainly extracted from brown algae is a fucose-enriched sulfated polysaccharide, and it has been widely used as a dietary supplement and health food due to its numerous beneficial effects, including anti-inflammatory, anticancer, and antidiabetic activities [17]. Clinical studies have indicated that administration of Haikun Shenxi capsule (the main active component is fucoidan) could markedly improve the clinical symptoms of patients suffering from chronic renal diseases or DN [18]. Accordingly, fucoidan may be a useful polysaccharide in the treatment of kidney diseases. Interestingly, different sulphate amounts and molecular weights of fucoidan, and the brown algae species used, are all important factors determining the functions of fucoidan, and low molecular weight fucoidan is thought to have better activity [19]. Although fucoidan is reported to alleviate DN in spontaneous diabetic mice [20], the true mechanisms involved are still poorly understood and require further elucidation. In this study, we examined whether low-molecular-weight fucoidan (oligo-FO) has a protective effect against renal fibrosis and dysfunction in diabetic mice induced by a high-fat diet and streptozotocin (STZ). Furthermore, the detailed molecular mechanisms underlying the actions of the oligo-FO were investigated with a focus on the role of Sirt-1, Nrf2/HO-1, and GLP-1R in AGE-stimulated rat renal proximal tubular epithelial cells (NRK-52E).

## 2. Materials and methods

### 2.1. Chemicals

The primary antibodies, including anti-Sirt-1, anti-RAGE, anti-GLP-1R, ubiquitin-specific peptidase 22 (anti-USP22), anti-transforming growth factor-β1 (TGF-β1), anti-TGF-β1R, anti-Smad 2/3, anti-FN, anti-high mobility group box 1 (HMGB1), anti-HO-1, anti-Keap1, anti-Nrf2, anti-nuclear factor-kappa B (NF-κB) p65, anti-HIF-1α, anti-histone 1.4, and anti-α-tubulin, were purchased from Abcam (Cambridge, MA, USA). Other primary antibodies of AMP-activated protein kinase (AMPK) and phospho-AMPK were purchased from Cell Signaling Technology (Danvers, MA, USA). The horseradish peroxidase (HRP)-labeled secondary antibody, goat anti-mouse IgG-biotin secondary antibody, and goat anti-rabbit IgG-biotin secondary antibody were obtained from Santa Cruz Biotechnology (Santa Cruz, CA, USA). STZ, nicotinamide, EX527, a selective inhibitor of Sirt-1/Sirt-2, tin protoporphyrin IX (SnPP), an inhibitor of HO-1, ED9-39, a selective inhibitor of GLP-1R, and other reagents and chemicals were all purchased from Sigma (Saint Louis, MO, USA). The oligo-FO from Sargassum Hemiphyllum was kindly provided by Hi-Q Marine Biotech International Ltd (Taipei, Taiwan), and it was prepared as described previously [21]. Briefly, the lyophilized hot water extract was incubated with 95% ethanol overnight at 4 °C followed by centrifugation and lyophilization to collect the crude extract sample. Then, the sample was suspended in distilled water and glycolytic enzyme (1 mg/g sample) was added for 6 h. After centrifugation at 10,000× g for 20 min at 4 °C, the supernatant was passed through series molecular weight cut-off membranes to obtain the oligo-FO with average molecular weight of 800 Da, fucose 210.9 ± 3.3 μmol/g, and sulfate 38.9 ± 0.4% (*w*/*w*). The oligo-FO was dissolved in distilled H_2_O and stored at 4 °C until use.

### 2.2. Cell Culture

The NRK-52E cells purchased from ATCC (NO. CRL-1571™, Taipei, Taiwan) were grown in Complete Dulbecco Minimum Essential Medium (DMEM) supplemented with 10% fetal bovine serum in an incubator at 37 °C with 5% CO_2_ and 95% humidity.

### 2.3. Cell Viability Assay

The MTT [3-(4,5-dimethyl-2-thia-zolyl)-2,5-diphenyl-2-*H*-tetrazolium bromide] assay was used to determine the viability of NRK-52E cells. After cells were treated with various agents for 24 h followed by addition of MTT (0.5 mg/mL) for 2 h, the absorbance at 570 nm was measured with a microplate reader.

### 2.4. Sirt-1 Activity Measurement

The Sirt-1 activity was determined by using Sirt-1 Combo Transcription Factor Assay Kit (Abcam, Ann Arbor, MI, USA).

### 2.5. Western Blotting Assay

The protein samples (50–100 μg) from cells and kidney tissues were separated on 10% sodium dodecyl sulfate (SDS)/polyacrylamide gel and transferred onto nitrocellulose membranes. Then, the membranes were incubated in Tris-buffered saline with 0.1% Tween 20 (TBST) containing 5% nonfat milk for 1 h at room temperature, followed by addition of target primary antibodies, and incubated overnight at 4 °C. After washing with 5% TBST, the secondary antibody was added and incubated for 1 h at room temperature, and the protein bands were detected by using enhanced chemiluminescence reagent (Milipore, Billerica, MA, USA). The α-Tubulin was used as the internal control.

### 2.6. Co-Immunoprecipitation (Co-IP) Assay

The anti-Sirt-1 or anti-HMGB1 antibody were incubated with cells. After rocking for 24 h at 4 °C, protein A magnetic beads (Millipore Corporation, Billerica, MA, USA) were added for precipitation. The eluted proteins were separated on 10% SDS-polyacrylamide gel, and the target protein was detected by Western blot analysis.

### 2.7. Animals and Treatment

The 6-week-old male C57BL/6 mice purchased from National Laboratory Animal Center (Taipei, Taiwan) were divided into three groups at random (*n* = 8 in each group). The mice of normal group were fed with normal chow (LabDiet 5010, 5.5% fat). The mice of diabetic group were fed with high-fat diet (61.6% fat, HFD, 58Y1, DIO Rodent Purified Diet, TestDiet) for 8 weeks, followed by an intraperitoneal injection of STZ (50 mg/kg in citrate phosphate buffer) and nicotinamide (200 mg/kg) on seven consecutive days to induce DN. The mice of normal group were injected with saline. Our preliminary data showed that administration of oligo-FO (300 mg/kg BW) had better effects on improvement of renal functions in the diabetic mice than that of oligo-FO (150 mg/kg BW). Moreover, the effects of oligo-FO (600 mg/kg BW) were similar to that of oligo-FO (300 mg/kg BW) (Figure S1). Thus, the dose of oligo-FO at 300 mg/kg BW was used for the in vivo study. The FO-treated diabetic mice were treated with oligo-FO (300 mg/kg/day) through oral gavage for 8 weeks. The animal experiments were approved by Animal Care and Use Committee, National Defense Medical Center, Taipei, Taiwan (# IACUC-15-215).

### 2.8. Biochemical Analysis and Histological Examination

The serum lipid profile and renal function markers were determined by routine procedure. After the renal tissues were fixed with 10% formaldehyde and embedded in paraffin, the sections (5 mm thick) were cut for hematoxylin and eosin (H&E) staining to evaluate the pathological changes. For immunohistochemical assay, the tissue samples were incubated with various primary antibodies overnight and secondary antibody (1:300, Abcam, MA, USA) was added for 1h and detected with diaminobenzidine peroxidase substrate and photographed.

### 2.9. Statistical Analysis

One-way ANOVA with a post hoc Bonferroni test was performed to analyze the data, and the difference was regarded as statistically significant when *p* values < 0.05. Data were expressed as mean ± standard error of the mean (S.E.M.). 

## 3. Results

### 3.1. Oligo-FO Increased Sirt-1 Expression and Activity, but Inhibited RAGE/NF-κB/TGF-β1/TGF-β1R/Smad 2/3/FN Cascade in AGE-Stimulated NRK-52E Cells

**Figure 1:**
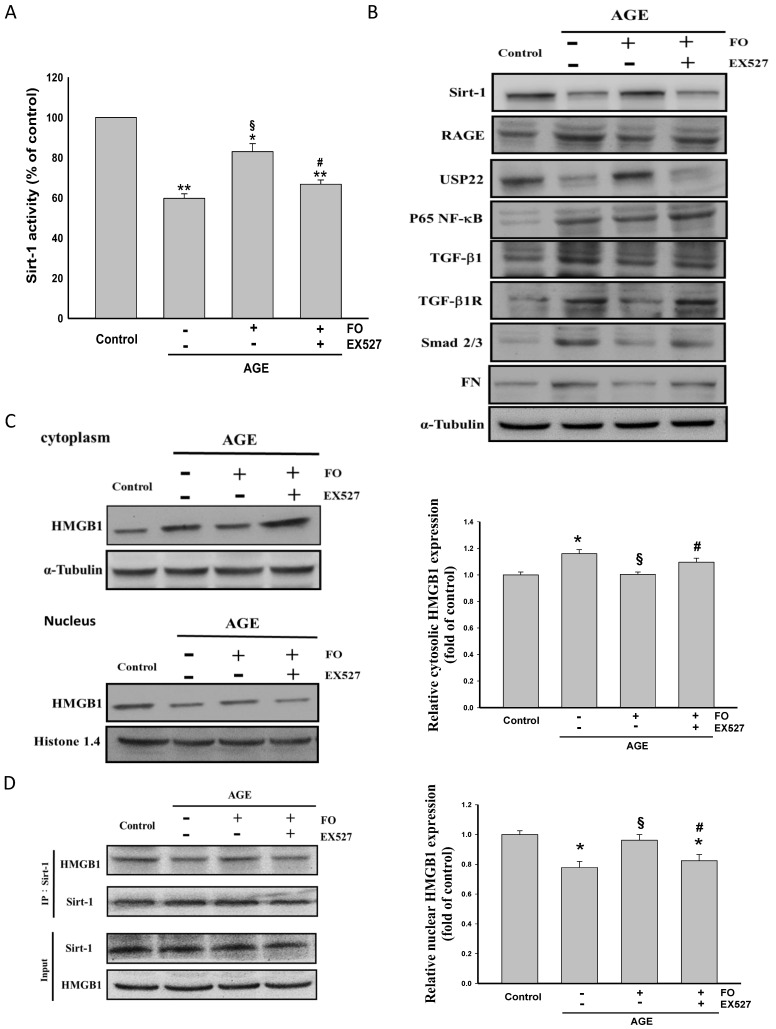
The effects of fucoidan on Sirt-1 activity, anti-high mobility group box 1 (HMGB1) cellular location, and fibrosis-related signaling pathways. The rat renal proximal tubular epithelial cells (NRK-52E) cells were incubated with advanced glycation product (AGE) (100 μg/mL) for 24 h followed by treatment with fucoidan (100 μg/mL) for 24 h in the presence or absence of EX527 (200 nM). The Sirt-1 activity (**A**), the expression of fibrosis-related genes (**B**), the cytosolic and nuclear levels of HMGB1 (**C**), and the association of Sirt-1 with HMGB1 in the nucleus (**D**) were determined in various groups. FO: fucoidan. Results were expressed as the mean ± SEM (*n* = 5). * *p* < 0.05, ** *p* < 0.01 vs. control group (untreated NRK-52E cells); ^§^ *p* < 0.05 vs. AGE-treated alone cells; ^#^ *p* < 0.05 vs. AGE and fucoidan-treated cells.

It has been confirmed that TGF-β1/TGF-βR/Smad 2/3-induced ECM generation leading to renal fibrosis plays a key role in the development of DN [3]. The interaction of AGE and its receptor, RAGE, and NF-κB activation are known to promote the transcription of TGF-β1 [22,23]. Importantly, Sirt-1 is able to inhibit TGF-β/Smad cascade [6]. A significant decrease in Sirt-1 expression and activity, downregulation of USP22 that can stabilize Sirt-l [24], and increased RAGE/NF-κB/TGF-β1/TGF-βR/Smad 2/3/FN signaling pathway were seen in AGE-stimulated NRK-52E cells, whereas oligo-FO markedly reversed these events (Figure 1A,B). Addition of AGE resulted in elevated cytoplasmic level of HMGB1, a ligand of RAGE, accompanied by lower nuclear amount of HMGB1 (Figure 1C), thereby decreasing the association of HMGB1 with Sirt-1 in the nucleus of NRK-52E cells (Figure 1D), which were significantly inhibited by oligo-FO. However, the effects of the oligo-FO were greatly diminished by EX527, an inhibitor of Sirt-1, indicating that the inhibitory effect of oligo-FO on the pro-fibrogenic pathway is modulated by Sirt-1.

### 3.2. Oligo-FO Enhanced AMPK and Nrf2 Activity and GLP-1R Expression

**Figure 2:**
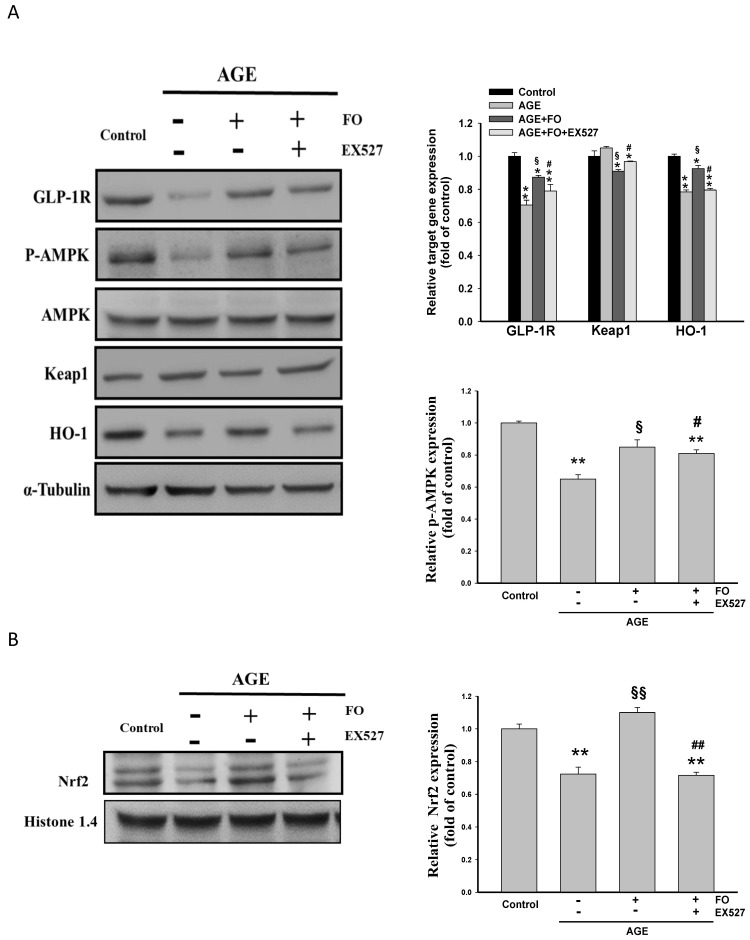
The effects of fucoidan on Nrf2 activation and the expression of glucagon-like peptide-1 receptor (GLP-1R), AMP-activated protein kinase (AMPK), and heme oxygenase-1(HO-1). The NRK-52E cells were incubated with AGE (100 μg/mL) for 24 h followed by treatment with fucoidan (100 μg/mL) for 24 h in the presence or absence of EX527 (200 nM). The expression of glucagon-like peptide-1 receptor (GLP-1R), AMP-activated protein kinase (AMPK), *p*-AMPK, Kelch-like ECH-associated protein 1 (Keap1), and HO-1 (**A**), and the nuclear level of Nrf2 (**B**) was determined in various groups. Results were expressed as the mean ± S.E.M (*n* = 5). * *p <* 0.05, ** *p* < 0.01 vs. control group; ^§^ *p* < 0.05, ^§§^ *p* < 0.01 vs. AGE-treated alone cells; ^#^ *p* < 0.05, ^##^ *p* < 0.01 vs. AGE and fucoidan-treated cells.

It is known that Nrf2/HO-1 cascade and GLP-1R exhibit potent anti-fibrotic activity, and AMPK is capable of increasing Sirt-1 transcriptional activity [10,14,25]. Our data showed that AGE induced obvious decline in the levels of GLP-1R and P-AMPK, which was attenuated by oligo-FO (Figure 2A). Oligo-FO treatment also enhanced Nrf2 activity, evidenced by an elevation in the nuclear level of Nrf2 (Figure 2B) and a reduction of cytosolic Keap1 expression accompanied by increased expression of HO-1 in AGE-stimulated cells (Figure 2A). Similarly, these effects of oligo-FO were significantly inhibited by EX527, suggesting that Sirt-1 positively regulates the expression of GLP-1R and P-AMPK, and Nrf2 activity.

### 3.3. Involvement of HO-1on Oligo-FO-Mediated Responses

**Figure 3:**
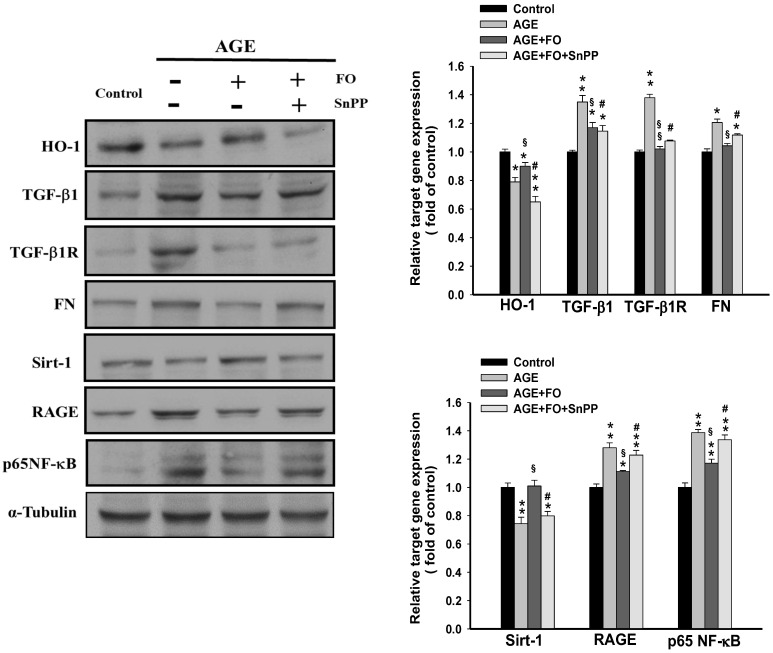
The effects of tin protoporphyrin IX (SnPP) on fucoidan-regulated target gene expression. The NRK-52E cells were incubated with AGE (100 μg/mL) for 24 h, followed by treatment with fucoidan (100 μg/mL) for 24 h in the presence or absence of SnPP (20 μM). The alterations of target gene expression were determined in various groups. Results were expressed as the mean ± S.E.M (*n* = 5). * *p <* 0.05, ** *p* < 0.01 vs. control group; ^§^ *p* < 0.05, ^§§^ *p* < 0.01 vs. AGE-treated alone cells; ^#^ *p* < 0.05 vs. AGE and fucoidan-treated cells.

As shown in Figure 3, blocking HO-1 activity with SnPP markedly reversed oligo-FO-regulated inhibition of RAGE/TGF-β1/TGF-β1R/FN cascade and NF-κB activation, and increased Sirt-1 expression in AGE-stimulated cells. Thus, activation of Nrf2/HO-1 may be involved in the suppression of RAGE/TGF-β1/TGF-β1R/FN pathway by oligo-FO.

### 3.4. Involvement of GLP-1R on Oligo-FO-Mediated Responses

**Figure 4:**
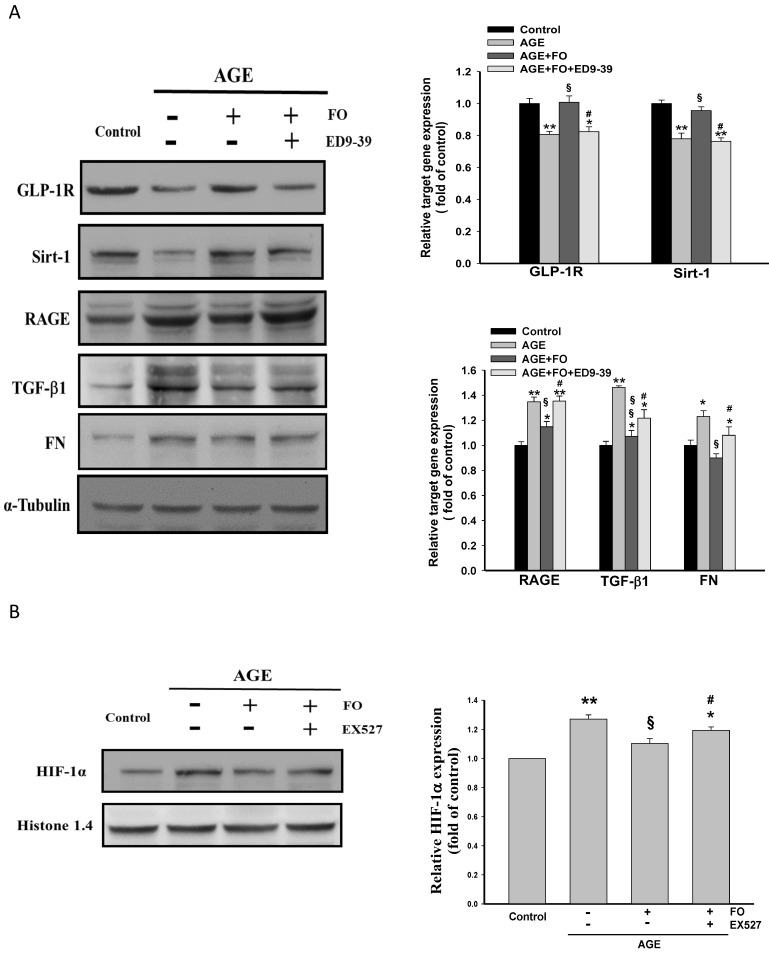
The effects of exendin-3 (9-39) (ED9-39) on fucoidan-regulated target gene expression. The NRK-52E cells were incubated with AGE (100 μg/mL) for 24 h followed by treatment with fucoidan (100 μg/mL) for 24 h in the presence or absence of ED9-39 (1 mM). The protein expression of target genes (**A**), and the nuclear level of hypoxia-inducible factor-1α (HIF-1α) (**B**), were determined in various groups. Results were expressed as the mean ± S.E.M (*n* = 5). * *p* < 0.05, ** *p* < 0.01 vs. control group; ^§^ *p* < 0.05, ^§§^ *p* < 0.01 vs. AGE-treated alone cells; ^#^ *P* < 0.05 vs. AGE and fucoidan-treated cells.

A novel finding of the present study was that oligo-FO is able to enhance GLP-1R level in AGE-stimulated NRK-52E cells. To further understand the role of GLP-1R, a selective *GLP-*1R** antagonist, exendin-3 (9-39) (ED9-39), was added. Similarly, the inhibition of RAGE/TGF-β1/FN cascade and the induction of Sirt-1 by oligo-FO were significantly reduced by ED9-39 (Figure 4A), indicating that there is a positive regulatory loop between GLP-1R and Sirt-1. Accordingly, *GLP-*1R** is an important mediator modulating the anti-profibrogenic activity of oligo-FO. The hypoxia-inducible factor-1α (HIF-1α), a transcription factor, is reported to induce RAGE [26]. Our results showed that AGE-induced nuclear translocation of HIF-1α was significantly inhibited by oligo-FO, whereas it was diminished by EX527 (Figure 4B), suggesting that the inhibition of HIF-1α activation may be also regulated by Sirt-1.

### 3.5. Oligo-FO Improved Renal Histological Changes and Dysfunction in the Diabetic Mice

**Figure 5:**
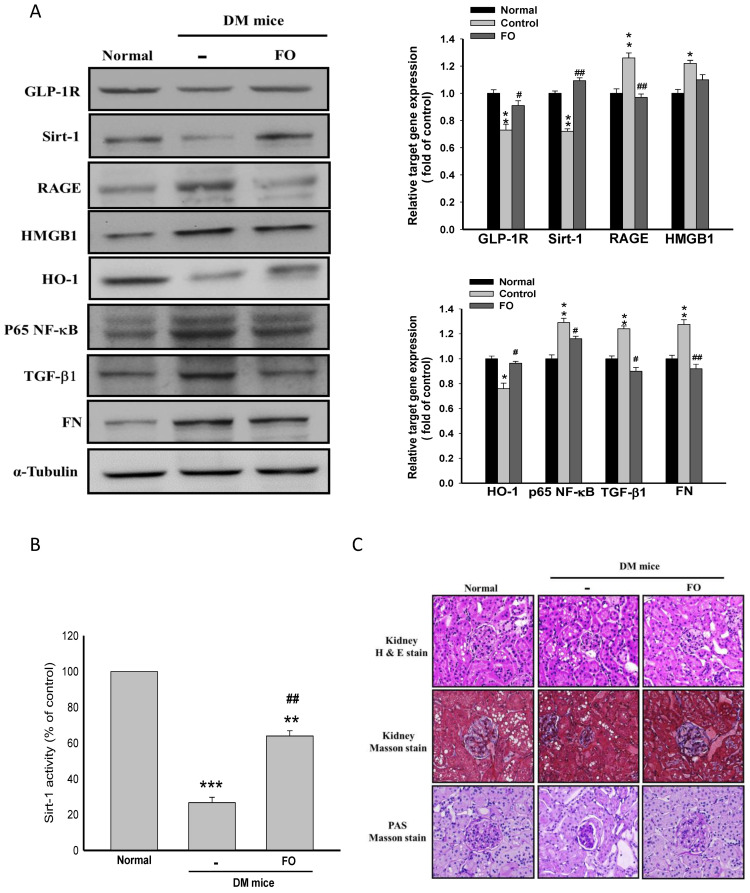
The effects of fucoidan on fibrosis-related gene expression, Sirt-1 activity, and the histological changes in the kidney of diabetic nephropathy mice. The expression of target genes (**A**), the Sirt-1 activity (**B**), and the histological changes evaluated by Masson staining and PAS staning (**C**) in the kidney of the diabetic mice were examined in different groups. Results were expressed as the mean ± SEM (*n* = 8). * *p* < 0.05, ** *p* < 0.01, *** *p* < 0.001 vs. normal mice; ^#^ *p* < 0.05, ^##^ *p* < 0.01 vs. untreated diabetic nephropathy mice.

**Figure 6:**
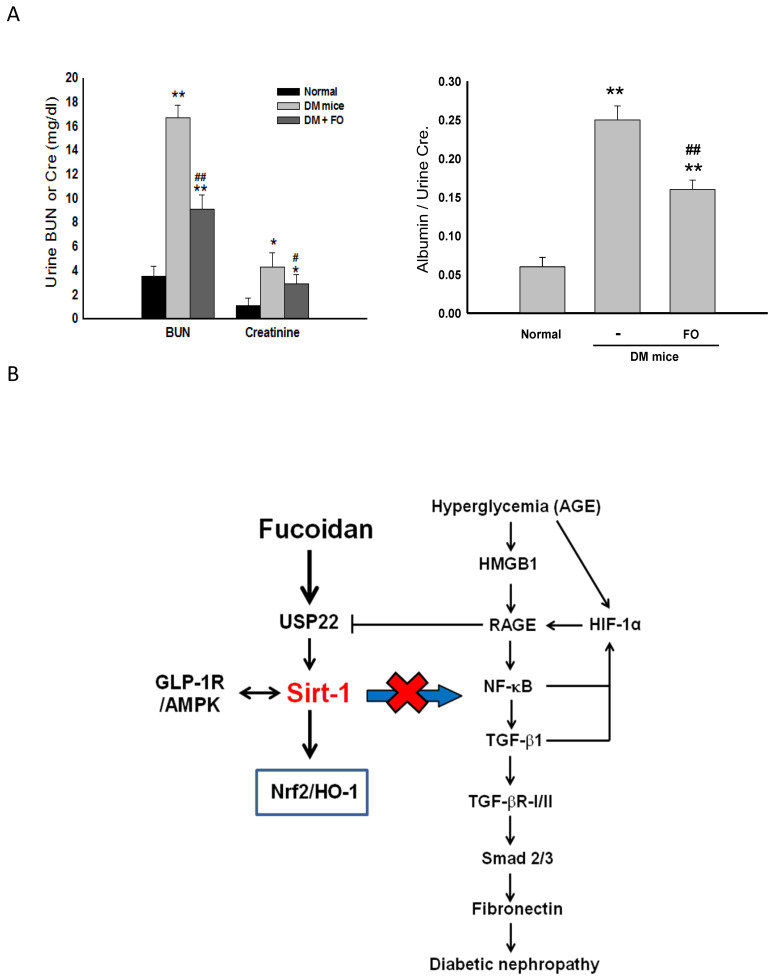
The effects of fucoidan on urine levels of BUN, creatinine, and albumin, in diabetic nephropathy mice. After treatment with fucoidan (1) (300 mg/kg, p.o.) for 6 weeks, the urine levels of BUN, creatinine, and albumin were examined in different groups (**A**). Results were expressed as the mean ± SEM (*n* = 8). * *p* < 0.05, ** *p* < 0.01 vs. normal mice; ^#^ *p* < 0.05, ^##^ *p* < 0.01 vs. untreated diabetic mice. The proposed schematic diagram of the protective effect of fucoidan against diabetic nephropathy (**B**).

In accordance with these results obtained from the in vitro study, administration of oligo-FO effectively increased the protein levels of Sirt-1, GLP-1R, and HO-1, as well as Sirt-1 activity, but inhibited NF-κB activation and HMGB1/RAGE/TGF-β1/FN cascade in the kidney of diabetic mice (Figure 5A,B). As expected, the renal histological changes and dysfunction evidenced by a significant elevation in the urine levels of BUN, creatinine, and albumin that occurred in the diabetic mice were markedly improved by oligo-FO treatment (Figure 5C and Figure 6A).

## 4. Discussion

DN is characterized by renal fibrosis and glomerular sclerosis, and it is closely linked to the occurrence of end-stage renal failure. Since current therapeutic drugs for DN are still unable to effectively attenuate and even delay the progression of DN, development of more effective agents for DN therapy is extremely important. In the present study, we demonstrated that the oligo-fucoidan greatly improves renal fibrosis under diabetic condition through inhibition of TGF-β1-activated pro-fibrogenic pathway via activation of Sirt-1, GLP-1R, and Nrf2/HO-1.

It is believed that TGF-β1 is a critical pathological factor in the pathogenesis of DN. When TGF-β1, the most abundant isoform presenting in all types of renal cells [3], binds to its receptor, TGF-βR, the downstream Smad 2 and Smad 3, are phosphorylated and the heteromeric complex with Smad 4 is formed, which subsequently translocates into nucleus where it promotes ECM related component transcription such as FN and collagens [27]. In addition, reduction of ECM degradation due to inhibition of matrix metalloprotenases (MMPs) activity is thought to be a mechanism contributing to TGF-β-induced fibrosis [28]. Under hyperglycemic condition, the AGE/RAGE axis can initiate the pathogenesis of DN through upregulation of TGF-β1 [29]. As expected, blocking TGF-β1/TGF-βR/Smad2/3 cascade remarkably attenuated kidney fibrosis in vitro and in vivo [30]. It has been reported that the protective effect of Sirt-1 against DN may attribute to inhibition of TGF-β/Smad cascade, inflammatory responses, renal fibrosis, and podocyte apoptosis, as well as prevention of mitochondrial dysfunction [31]. Clinical study further indicated that lower level of Sirt-1 is closely related to the occurrence and progression of DN [32]. Thus, suppressing AGE/RAGE/TGF-β1-mediated pro-fibrotic responses, and upregulation of Sirt-1and activation of its regulated processes, are promising strategy to attenuate renal fibrosis and DN progression. Our results revealed that the oligo-FO significantly reduced FN generation in AGE-stimulated NRK-52E cells, which may result from inhibition of RAGE/NF-κB/TGF-β1/TGF-βR/Smad 2/3/FN signaling pathway and increased expression and activity of Sirt-1. However, the events of oligo-FO were markedly diminished by EX527, which highlights the importance of Sirt-1 on the actions of oligo-FO.

Next, we explored how oligo-FO enhances Sirt-1 protein expression and activity. USP22, an ubiquitin-specific protease, is able to enhance Sirt-1 protein stability by preventing Sirt-1 degradation [24]. In contrast, AGE-RAGE system can decrease Sirt-1 protein level due to elevation of Sirt-1 degradation via downregulation of USP22 [33]. Interestingly, Sirt-1 also activates USP22 expression through activation of c-Myc [34], suggesting that a positively regulatory loop exists between Sirt-1 and USP22. Additionally, AMPK-triggered nuclear translocation of GAPDH leads to Sirt-1 release from the complex deleted in breast cancer-1 (DBC-1), an inhibitor of Sirt-1, thereby enhancing Sirt-1 transcriptional activity [25]. Compared with untreated cells, the lower protein levels of USP22 and *p*-AMPK observed in AGE-stimulated NRK-52E cells were significantly reversed by oligo-FO; however, the events of oligo-FO were abolished by EX527. Therefore, preventing Sirt-1degradation via upregulation of USP22 and activation of AMPK may contribute to oligo-FO-induced Sirt-1 protein level and activity.

In response to AGEs, HMGB1, a nuclear DNA-binding protein, is transferred from nucleus into the extracellular region and then binds to RAGE, which in turn further enhances HMGB1 and RAGE transcription via activation of NF-κB [35]. Notably, the release of HMGB1 is inhibited by Sirt-1through suppression of acetylation-triggered dissociation of HMGB1 from Sirt-1 in the nucleus [36]. A higher cytosolic HMGB1 protein level and lower nuclear HMGB1 protein level accompanied by a decrease in the interaction of HMGB1 and Sirt-1 in the nucleus found in AGE-stimulated cells were inhibited by oligo-FO, whereas it was diminished by addition of EX527. Thus, the beneficial effects of oligo-FO may be, at least partly, regulated by Sirt-1-dependent reduction of nuclear-to-cytoplasmic translocation of HMGB1.

The Nrf2/HO-regulated processes are regarded as a crucial adaptive system for maintaining cellular redox homeostasis and enhancing cellular resistance to oxidative stress [37]. Previous study has reported that the inhibitory effects of Nrf2 on pro-fibrogenic signaling pathway and DN progression in AGE-stimulated GMCs and STZ-induced diabetic mice are associated with downregulation of TGF-β1 and activation of Sirt-1 [38]. Our results showed that oligo-FO could significantly restore AGE-induced reduction of Nrf2 activation caused by AGE, evidenced by a marked elevation in the nuclear level of Nrf2 and a reduction of cytosolic Keap1 level accompanied by increased HO-1 expression. In the presence of EX-527 or SnPP, the effects of oligo-FO on Nrf2 activation, the expression of Sirt-1 and HO-1, and the fibrotic signaling pathway were all dramatically inhibited. These results suggest that there is a crosstalk between Sirt-1 and Nrf2/HO-1 cascade, and that Sirt-1/Nrf2/HO-1 cascade is involved in the anti-fibrogenic activity of oligo-FO.

Several basic and clinical studies have demonstrated that treatment with GLP-1 analogs or GLP-1R agonists greatly mitigates renal fibrosis associated with DN by inhibiting TGF-β1-activated Smad 3 and ERK1/2 [39]. Furthermore, GLP-1-activated cyclic AMP-protein kinase A is thought to increase Sirt-1 activity through phosphorylation of Sirt-1 (serine 434) [40,41]. We found that the oligo-FO also has an ability to enhance GLP-1R expression in AGE-stimulated NRK-52E cells, suggesting that oligo-FO may be a GLP-1R agonist. Similarly, co-treatment with ED9-39, an antagonist of GLP-1R, or EX527 could abolish the alterations of the expression of Sirt-1, GLP-1R, respectively, and the pro-fibrogenic signaling pathway by oligo-FO. These findings suggest that GLP-1R and Sirt-1 exert their functions via Sirt-1 and GLP-1R dependence. HIF-1α is a key transcription factor, and it can promote the pathogenesis of renal diseases such as DN through upregulation of RAGE and NF-κB activation [42]. Notably, TGF-β1 is capable of enhancing HIF-1α protein stability by preventing HIF-1α degradation via inhibition of prolyl hydroxylase 2 (PHD 2) activity [43]. In contrast, Sirt-1 inhibits HIF-1α1 activity through deacetylation of HIF-1α [44]. Accordingly, oligo-FO-mediated suppression of AGE-induced nuclear translocation of HIF-1α may be modulated by Sirt-1-dependent responses, which was supported by the result that the inhibition of HIF-1α activation by oligo-FO was attenuated by EX527. Collectively, Sirt-1-induced GLP-1R expression and HIF-1α inactivation may be another mechanisms contributing to the beneficial effects of oligo-FO.

To evaluate the therapeutic effects of oligo-FO in vivo, the alterations of these target gene expressions, and the renal morphology and functions after oligo-FO treatment, were examined in the diabetic mice. As expected, administration of oligo-FO (300 mg/kg BW) markedly alleviated the abnormalities of renal morphology and renal function occurred in the diabetic mice. Consistent with the results obtained from in vitro study, similar changes of the expression and activity of these target genes in the kidneys of the diabetic mice were seen after oligo-FO treatment.

## 5. Conclusions

We demonstrated that oligo-FO exerts protective effects against renal fibrosis and dysfunction associated with diabetes. Moreover, the present study may provide novel insights into the underlying mechanisms and indicate that suppressing the HMGB1/RAGE/NF-κB/TGF-β1/TGF-β1R/FN signaling pathway and HIF-1α activation via Sirt-1, GLP-1R, and Nrf2/HO-1 dependence may contribute to the therapeutic effects of oligo-FO on DN (Figure 6B). These findings supported that the oligo-FO is a potential agent to attenuate renal fibrosis in the diabetic animal model. To establish its clinical applications, more clinical trials are needed to confirm the safety, other potential effects, and the utilities of the oligo-FO on alleviating excessive fibrosis-associated renal diseases in patients with diabetes.

## Supplementary Materials

The following are available online at https://www.mdpi.com/2072-6643/12/10/3068/s1, Figure S1: The effects of various doses of fucoidan on renal function markers in the diabetic mice.

## Abbreviations

AGEs: advanced glycation products; AMPK, AMP-activated protein kinase; DN, diabetic nephropathy; FN, fibronectin; *FO, fucoidan;* GLP-1R, glucagon-like peptide-1 receptor; HIF-1α, hypoxia-inducible factor-1α; HMGB1, high mobility group box 1; HO-1, heme oxygenase-1; NF-κB, nuclear factor-kappa B; Nrf2, nuclear factor erythroid-2-related factor 2; ROS, reactive oxygen species; Sirt-1, sirtuin 1; STZ, streptozotocin; TGF-β1, transforming growth factor-β1; USP22, ubiquitin-specific peptidase 22.
