# Supplementary material for: Oligo-Fucoidan Improves Diabetes-Induced Renal Fibrosis via Activation of Sirt-1, GLP-1R, and Nrf2/HO-1: An In Vitro and In Vivo Study
Source: Nutrients. 2020 Oct 8;12(10):3068. doi: 10.3390/nu12103068 (PMC7650749; doi:10.3390/nu12103068)
Supplement: Supplementary file 1 [file nutrients-12-03068-s001.docx]

**Supplementary Materials**

**Figure S1.** The effects of various doses of fucoidan on renal function markers in the diabetic mice. After treatment with fucoidan (150-600 mg/kg, p.o.) for 6 weeks, the urine levels of BUN, creatinine, and albumin were examined in different groups. The values were expressed as the mean ± SEM (n = 8). **P* < 0.05, ***P* < 0.01 vs. untreated diabetic mice.
